# Low-density Lipoprotein Receptor-related Proteins in a Novel Mechanism of Axon Guidance and Peripheral Nerve Regeneration*

**DOI:** 10.1074/jbc.M115.668996

**Published:** 2015-11-23

**Authors:** Lila M. Landowski, Macarena Pavez, Lachlan S. Brown, Robert Gasperini, Bruce V. Taylor, Adrian K. West, Lisa Foa

**Affiliations:** From the ‡ School of Medicine and; § Menzies Institute for Medical Research, University of Tasmania, Hobart, Tasmania 7001, Australia

**Keywords:** axon, cell signaling, neurite outgrowth, neurobiology, regeneration, LRP1, LRP2, metallothionein, growth cone

## Abstract

The low-density lipoprotein receptor-related protein receptors 1 and 2 (LRP1 and LRP2) are emerging as important cell signaling mediators in modulating neuronal growth and repair. We examined whether LRP1 and LRP2 are able to mediate a specific aspect of neuronal growth: axon guidance. We sought to identify LRP1 and LRP2 ligands that could induce axonal chemoattraction, which might have therapeutic potential. Using embryonic sensory neurons (rat dorsal root ganglia) in a growth cone turning assay, we tested a range of LRP1 and LRP2 ligands for the ability to guide growth cone navigation. Three ligands were chemorepulsive: α-2-macroglobulin, tissue plasminogen activator, and metallothionein III. Conversely, only one LRP ligand, metallothionein II, was found to be chemoattractive. Chemoattraction toward a gradient of metallothionein II was calcium-dependent, required the expression of both LRP1 and LRP2, and likely involves further co-receptors such as the tropomyosin-related kinase A (TrkA) receptor. The potential for LRP-mediated chemoattraction to mediate axonal regeneration was examined *in vivo* in a model of chemical denervation in adult rats. In these *in vivo* studies, metallothionein II was shown to enhance epidermal nerve fiber regeneration so that it was complete within 7 days compared with 14 days in saline-treated animals. Our data demonstrate that both LRP1 and LRP2 are necessary for metallothionein II-mediated chemotactic signal transduction and that they may form part of a signaling complex. Furthermore, the data suggest that LRP-mediated chemoattraction represents a novel, non-classical signaling system that has therapeutic potential as a disease-modifying agent for the injured peripheral nervous system.

## Introduction

During development, neuronal growth cones use a range of guidance cues to navigate the embryonic environment, establishing the early framework of the neuronal circuitry (1). Although the major families of guidance cues, such as netrins, ephrins, and semaphorins, are well established (reviewed in Ref. 1), it is likely that other context-dependent guidance cues exist. For example, the extracellular environment associated with growth cones in the developing nervous system is likely to be very different to that encountered by regenerating neurons during neuropathy or following physical injury in the mature brain (2). This raises the question whether other receptor-ligand signaling complexes outside of those established in neurodevelopment might be exploited in neuronal regeneration.

Low-density lipoprotein receptor-related protein (LRP)^3^ receptors LRP1 and LRP2 are the largest and most complex members of the low-density lipoprotein receptor family and are thought to play multiple roles in neuronal function (3, 4). The LRP receptors are highly promiscuous, binding a wide range of functionally distinct ligands, several of which have established roles in the nervous system, including apolipoprotein E3 (ApoE3), sonic hedgehog, myelin-associated glycoprotein, tissue plasminogen activator (tPA), and metallothionein II (MT I/II) (5–9). At the cellular level, LRP1 and LRP2 are required for the development of neuronal and glial precursor cells (10, 11). In knockout mouse models, LRP1 deficiency is lethal early in development (12), whereas neuron-specific LRP1 cre-lox mice exhibit severe tremor and dystonia, behavioral abnormalities, hyperactivity, age-dependent dendritic spine degeneration, synapse loss, neuroinflammation, memory loss, eventual neurodegeneration, and premature death (13, 14). Knockout of LRP2 is similarly devastating, with gross forebrain defects and ventricle and choroid plexus malformation (3, 6). The resultant holoprosencephalic syndrome is similar to that seen in humans deprived of cholesterol during development (3). LRP2 mutation in humans results in Donnai-Barrow syndrome, an autosomal recessive disorder that disrupts brain formation (15).

LRP function has also been implicated in neuronal injury and disease. In Alzheimer disease, LRP1 has been shown to mediate the clearance of β-amyloid from the brain (reviewed in Ref. 4). LRP receptors have been shown to mediate axonal regeneration after injury (16–18). Activation of LRP1 and LRP2 post-injury by MT I/II promotes neurite sprouting of central and peripheral neurons (17). Given the emerging role of LRPs in modulating neuronal growth during development and injury, we hypothesized that LRP1 or LRP2 could mediate chemotaxis and guide extending axons *in vitro* and *in vivo* after axonal injury.

## Experimental Procedures

### Primary DRG Neuron Culture

All animal experimentation was performed with approval from the University of Tasmania Animal Ethics Committee and complied with the Australia Code for the Care and Use of Animals for Scientific Purposes.

Sensory neurons were cultured from embryonic days 16–18 dorsal root ganglia (DRG) from Sprague-Dawley rats, as described previously (19). Briefly, thoracolumbar DRG were dissected into sensory neuron medium containing Dulbecco's modified Eagle's F-12 medium (1:1), penicillin G (100 units/ml), streptomycin (100 μg/ml), and 1× N2 neural medium supplement (all from Gibco Biosciences), nerve growth factor (50 ng/ml, Sigma-Aldrich), and fetal calf serum (5% v/v, Bovogen Biologicals). Dissociated DRG cells were plated onto coverslips coated with laminin (50 μg/ml, Invitrogen) and poly-l-ornithine (1 mg/ml, Sigma-Aldrich) in 35-mm Petri dishes (Iwaki, Tokyo, Japan). Unless stated otherwise, cultures were grown at 37 °C, 5% CO_2_ for at least 2 h prior to imaging.

### In Vitro Growth Cone Turning Assay

The growth cone turning assay was performed as described previously (19, 20). The reagents used to generate the microgradient were as follows: LRP ligands MTII (the MTIIA isoform of MTII was used and will be referred to as MTII in the text; rabbit-derived, HPLC-purified, 300 μg/ml, Zn_7_ form, Bestenbalt LCC, Tallinn, Estonia), ApoE3 (1.8 mg/ml, R&D Systems, Minneapolis, MN), LRP receptor-associated protein (RAP, 1 mg/ml in Tris-buffered saline, a gift from David Small, University of Tasmania), α2-macroglobulin (α2m) from human plasma (2.25 mg/ml, Sigma-Aldrich), transthyretin (8 mg/ml, a gift from David Small), vitamin D (100 μmol/liter, Sigma-Aldrich) combined with a 1:1 molecular ratio of vitamin D binding protein (Abcam, Cambridge, UK), tPA (3.3 mg/ml, Abcam), Netrin-1 (5 μg/ml, R&D Systems), and zinc sulfate (50 mm, Sigma-Aldrich). The control microgradient was PBS (pH 7.4). The bath-applied pharmacological agents were as follows: RAP (25 μg/ml), Tris-buffered saline, KN93 (5 μm, Calbiochem), KN92 (5 μm, Calbiochem), thapsigargin (50 nm, Alomone Labs, Jerusalem, Israel), K252a (100 nm, Calbiochem), and GW441756 (Tocris Bioscience). Pharmacological agents or vehicle were added to the culture medium 20 min prior to imaging. The TrkA antibody (rabbit, binds the extracellular fragment of the rat TrkA receptor, amino acids 1–416, diluted 1:1000, Abcam) was also bath-applied. Cultures were preincubated in the antibody for 5 min prior to imaging.

For cultures grown in low-calcium medium, sensory neuron medium was removed immediately prior to imaging and replaced with Ca^2+^ free Hanks' balanced salt solution comprising Hank's balanced salt solution Ca^2+^- and Mg^2+^-free media (Gibco Biosciences) with penicillin G, streptomycin, nerve growth factor, and N2 neural medium supplement (as described above).

Time-lapse images were acquired by Matlab and Stimulink V7.1.0.124 (The Mathworks) every 7 s for 30 min. Axonal trajectories were measured using ImageJ (19). Axons that extended less than 10 μm were excluded from analysis. Statistical analysis was conducted in GraphPad Prism V4.03 (Software Mackiev). All significance values are the product of a Mann-Whitney *t* test (20).

### Cell Culture Immunocytochemistry

The antibodies used were as follows: rabbit LRP-1 antibody (diluted 1:1000, catalog no. L2170, Sigma-Aldrich), rabbit LRP-2 antibody (diluted 1:1000, catalog no. sc-H245, Santa Cruz Biotechnology), rabbit TrKA antibody (diluted 1:10,000, catalog no. ab8871, Abcam), and rabbit phosphorylated TrKA antibody (pTrKA, diluted 1:200, catalog no. ab1445, Abcam). Immunocytochemistry was performed as described previously (19). Briefly, DRG cultures were fixed with 4% paraformaldehyde (Sigma-Aldrich) after random growth for 2–6 h or after being exposed to a microgradient of MTII for 15 min and incubated in primary antibody overnight at 4 °C. Primary antibody was omitted in zero primary controls. After washing in PBS, cells were incubated in secondary antibody (Alexa Fluor 594/488, goat anti-rabbit, 1:1000, Molecular Probes). Some cultures were counterstained with Alexa Fluor 488-phalloidin (1:40 in PBS, Molecular Probes). Cultures were mounted with DPX mounting medium (Sigma-Aldrich). Images were acquired using either a spinning disc confocal microscope equipped with Volocity software (PerkinElmer Life Sciences) and a Nikon Eclipse T.I microscope using a ×40 Plan Apo lens (Nikon, Tokyo, Japan) or an Olympus BX50 microscope equipped with a UPlanSApo ×60 1.35 water immersion lens (Olympus, Tokyo, Japan) and captured with a cooled charge-coupled device camera (Coherent). Images were processed using ImageJ (21) and JASC Paint Shop Pro V 9.0 (JASC Software Inc.).

To analyze the distribution of proteins during turning, the cells were fixed and processed for immunocytochemistry as described above. Using ImageJ, a line extending from the axon through the growth cone was drawn through the actin image of the growth cone, dividing the growth cone into near and far with respect to micropipette position (Fig. 2, *D* and *E*). The protein immunoreactivity of the near and far sides was assessed by comparing integrated pixel intensity normalized to the area as described previously (19).

### LRP Knockdown

We used siRNA to reduce LRP protein in DRG neurons using a technique published previously (19). Four LRP1 siRNAs (GGGCAUUUGUGCUGGACGA, GGACAGACGUGACGACCCA, UCAAUAAGCAGACGGGAGA, and UGGACAAGAUCGAACGUAU) and four LRP-2 siRNAs (CCUCAGUUGACGACGAAUA, GAGGGAAAUCAGCGUGUUA, GGAACAUCUUCAAACGAAA, and GGAUGGUAGCAAUCGGAA) were trialed (all from Thermo Fisher Scientific). Individual specific or control siRNAs (50 μm) were loaded into neurons by trituration with the DRG prior to plating. Cultures were grown for a minimum of 6 h prior to imaging. Knockdown of LRP-1 and LRP-2 protein was quantified by immunofluorescence and Western blot analysis as described previously (19).

### Capsaicin-induced Denervation, Treatment, Imaging, and Quantification

#### 

##### Denervation

Anesthetized rats (2–3.5% isoflurane, 12 rats, male, 8 weeks old, ∼500 g) were shaved along the lumbosacral dorsum and treated with 8% capsaicin cream (Sigma-Aldrich) in emulsifying ointment B.P. (BiotechPharm) in a demarcated area, with vehicle control cream applied to the other side. The application regions were separated by 1.5 cm. After 60 min, the creams were removed.

##### Treatment

Six rats were allocated to either the saline or MTII treatment group. Anesthetized rats (2–3.5% isoflurane) were injected with either 0.3 mg/ml MTII (Zn_7_ form, Bestenbalt LCC) or 0.9% normal saline. Injections were made into both sides of the dorsum, which had been treated with vehicle or capsaicin cream. Injections were performed three times weekly for 2 weeks in the first 8 h after capsaicin treatment.

##### Biopsies

Punch biopsies (3-mm biopsy punch) were taken from both sides of the dorsum every 7 days for a total of 14 days. Rats were anesthetized (2–3.5% isoflurane). Tissue was immediately placed in Zamboni fixative for 3 h. Washed biopsies were placed in 30% sucrose until cryosectioning. Sections were permeabilized and blocked in 0.4% Triton X-100 (Sigma-Aldrich) supplemented with 5% goat serum. Sections were incubated overnight at 4 °C in primary antibody (βIII-tubulin, mouse, 1:1000, Promega). Controls were labeled with an IgG serum. Washed sections were incubated in DAPI (1:1000, Sigma-Aldrich) and secondary antibody (Alexa Fluor 594 goat anti-mouse, 1:1000, Molecular Probes) in the dark and overnight at 4 °C. Sections were dried and mounted in DPX mounting medium (Sigma-Aldrich).

##### Image Acquisition

Fluorescence images were acquired using Volocity software (PerkinElmer Life Sciences) with a Nikon Eclipse T.I confocal microscope equipped with a ×40 Plan Apo lens (Nikon). All images were captured with the same exposure and laser intensity at ×40 magnification. Images were processed using ImageJ (21) and JASC Paint Shop Pro V 9.0 (JASC Software Inc.). DAPI staining was used to identify the epidermal/dermal junction.

##### Nerve Density Analysis

Quantification of nerve density in punch biopsy samples was performed using ImageJ (21). Images of DAPI staining and βIII-tubulin immunoreactivity across all treatment groups were acquired. The operator was blind to the treatment. βIII-tubulin images were first background-corrected using a 5-pixel rolling ball radius. In the DAPI image, a line was drawn 7 μm above the basement membrane of the epidermis. A plot profile of the βIII-tubulin signal intensity along the line was acquired. Images were thresholded, and signal intensity above the threshold indicated the presence of a traversing immunoreactive nerve fiber. At least five fields per sample, at various locations within the biopsy, were processed for quantification.

### Statistical Analysis

Mann-Whitney *t* test was used to calculate the statistical significance for the growth cone turning assays. Student's *t* test was used for comparisons between control and knockdown protein levels in Western blot analyses and for the analysis of nerve density in skin biopsies.

## Results

### 

#### 

##### LRP2 and LRP1 Are Expressed in Growth Cones of Sensory Neurons

**FIGURE 1.**
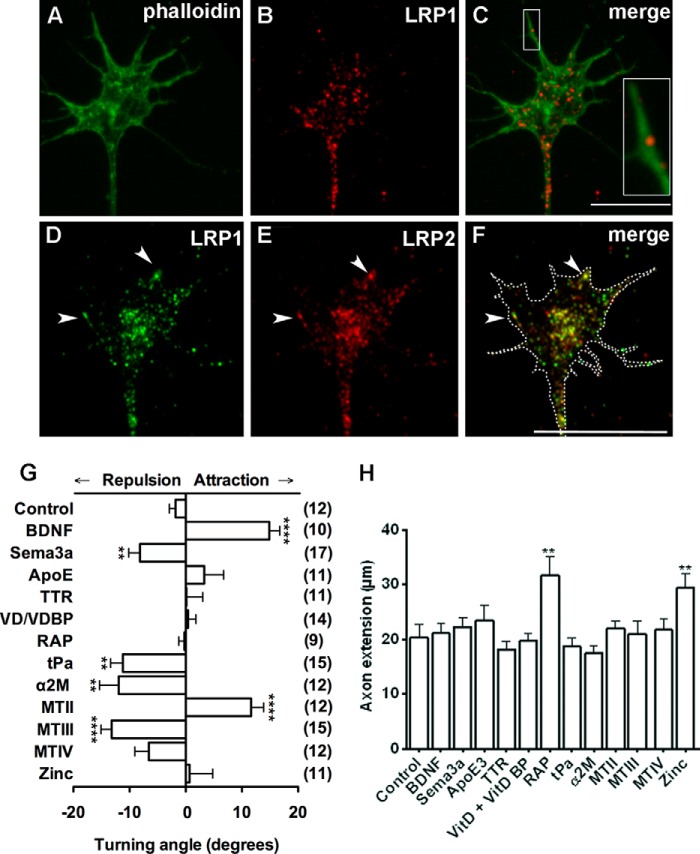
**LRP1 and LRP2 are expressed on growth cones, and LRP ligands can mediate growth cone navigation.** *A–C*, the structure of a DRG growth cone is highlighted by staining of filamentous actin (*green*, *A*). The same growth cone was stained for LRP1 (*red*, *B*). The merged image (*C*) highlights that LRP1 puncta can be found along the filopodia (*inset*). *D–F*, LRP1 and LRP2 expression is closely associated along the leading edge and within growth cones (*arrowheads*). The *dashed outline* in *F* highlights the periphery of the growth cone traced from the phase-contrast image (data not shown). *G*, LRP ligands can mediate growth cone chemotaxis. A microgradient of MTII was chemoattractive. Microgradients of α2m, tPA, and MTIII were chemorepulsive. *H*, the only LRP ligand to change axon extension was RAP. *Scale bars* = 10 μm (*C*, relates to *A–C*) and 10 μm (*F*, relates to *D–F*).****, *p* < 0.0001; **, *p* < 0.01. *TTR*: transthyretin.

Given the ability for LRP ligands to promote neurite outgrowth and survival during injury (18, 22), we asked whether LRP1 or LRP2 could mediate the guidance of sensory axons *in vitro*. If LRP1 and LRP2 are able to mediate axon guidance signals, then we would predict that they are expressed on growth cone membranes. Both LRP1 and LRP2 were expressed on sensory growth cones (Fig. 1). LRP1-immunoreactive puncta were evident throughout the growth cone (Fig. 1, *B* and *C*) and along the filamentous actin-rich filopodia (Fig. 1, *A–C*, *inset*). LRP2-immunoreactive puncta were also expressed throughout the growth cone, including the leading edge and filopodia (Fig. 1, *E* and *F*, *arrowheads*). No immunoreactivity was detected when the primary antibody was omitted (data not shown). Furthermore, LRP1 and LRP2 were often seen to be closely associated, suggesting co-localization (Fig. 1, *D–F*, *arrowheads*). Although neuronal expression of LRP1 and LRP2 has been demonstrated previously (14, 18), this work is the first to demonstrate LRP1 and LRP2 expression in sensory neuronal growth cones.

##### Ligands of LRP1 and LRP2 Are Chemotactic

Given that LRP1 and LRP2 are present in growth cones, we sought to determine whether any of the well characterized LRP1 and LRP2 ligands could exert chemotactic effects on growth cones. We tested the ligands α2m, ApoE3, tPA, vitamin D/vitamin D binding protein complexes (VD/VDBP), transthyretin, RAP, MTII, and the related proteins MTIII and MTIV in a growth cone turning assay (20). A gradient of ApoE3, transthyretin, VD/VDBP, RAP, or MTIV resulted in random turning, not different from the control gradient of PBS. Only four LRP ligands elicited significant growth cone turning, and, of those, MTII was the only LRP ligand to elicit growth cone attraction (11.6° ± 2.3°, *p* < 0.001, Fig. 1*G*). The turning angle in response to MTII was comparable with DRG turning in response to BDNF (Fig. 1*G*), a known DRG growth cone chemoattractant (19). Because MTII was used in its zinc-bound form, it was important to assess whether a zinc solution (zinc sulfate, 50 mm) alone exerted any directional influence. However, zinc alone did not cause any chemotactic effect (Fig. 1*G*). Three ligands induced growth cone repulsion: α2m induced robust growth cone repulsion compared with the control (−11.9° ± 3.4° *versus* −1.1° ± 1.8°, *p* < 0.01, Fig. 1*G*), tPA was chemorepulsive (−11.1°± 2.1°, *p* < 0.001), and, interestingly, MTIII, a related protein very similar in structure to MTII, was also chemorepulsive (−13.2° ± 1.9°, *p* < 0.0002, Fig. 1*G*). This is consistent with the neurite growth inhibition effects of MTIII reported previously (23). The turning angles induced by α2m, tPA, and MTIII were comparable with DRG turning in response to Semaphorin 3a (Fig. 1*G*), known to be chemorepulsive to DRG growth cones (24). To ensure that any effect of the LRP ligands on growth cone turning was due to chemotaxis and not altered growth patterns, axon extension was measured during the 30 min of imaging (Fig. 1*H*). Most LRP ligands did not alter axon extension during the 30-min imaging period, although axon extension was enhanced in response to the gradient of RAP and zinc, which is consistent with previous studies (25, 26).

##### MTII-mediated Growth Cone Chemoattraction Is Dependent on LRP1 and LRP2

**FIGURE 2.**
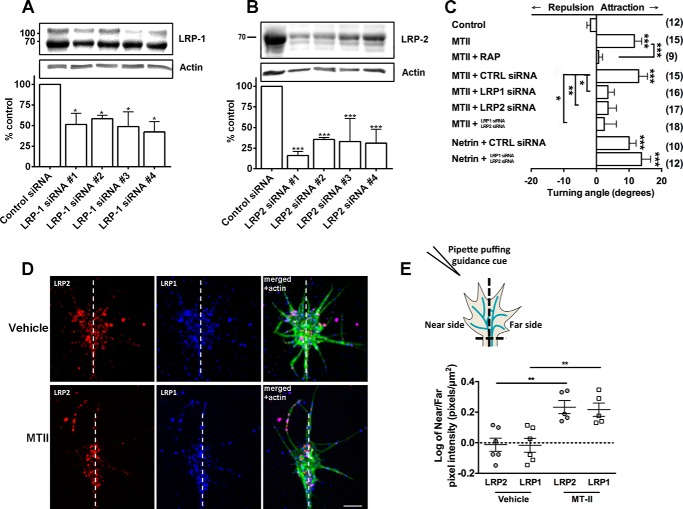
**LRP1 and LRP2 expression are required for MTII-mediated chemoattraction.** *A*, Western blot analysis of DRG cultures demonstrating that four specific LRP1 siRNA oligonucleotides reduced LRP1 expression to ∼50% of control levels. *B*, Western blot analysis demonstrating that four specific LRP2 siRNA oligonucleotides reduced LRP2 expression to ∼29% of control levels. *C*, growth cone navigation toward a microgradient of MTII was abolished by the pan-LRP inhibitor RAP. Reduction of LRP1 or LRP2 expression also abolished navigation toward MTII, as did simultaneous knockdown of LRP1 and LRP2. Reduction of LRP1 and LRP2 expression had no effect on growth cone navigation toward a microgradient of Netrin-1. *CTRL*, control. *D*, representative immunocytochemistry images of individual growth cones turning in response to microgradients of vehicle (PBS) or MTII. Growth cones were rapidly fixed during turning and then stained for LRP1 (*blue*) or LRP2 (*red*) and actin (*green*). The actin labeling was used to depict the growth cone area, and the growth cones were divided into near and far regions with respect to the micropipette for pixel intensity analysis. The *dotted line* drawn from the axon through the growth cone separates the near and far regions of the growth cone. The near side is the left side of the growth cone, as depicted in *E. Scale bar* = 10 μm (applies to all panels). *E*, schematic demonstrating how the near and far sides of the growth cone were divided and resulting quantification of LRP1 and LRP2 distribution within the growth cone. ***, *p* < 0.0002; **, *p* < 0.01; *, *p* < *0.05*.

Novel chemoattractive guidance cues have potential therapeutic benefits. Therefore, for the remainder of this work, we focused on the mechanisms underlying MTII-LRP mediated growth cone chemoattraction. To determine which specific LRP receptors are responsible for mediating MTII-induced growth cone attraction, we used siRNA oligonucleotides to reduce endogenous expression of LRP1 and LRP2, the known receptors of MTII. We used four different siRNAs each for both LRP1 and LRP2 receptor protein knockdown. The level of protein knockdown in DRG cultures was assayed by Western blot analysis and quantified relative to actin loading controls (Fig. 2, *A* and *B*). For both LRP1 and LRP2, all four siRNAs yielded equally significant knockdown of protein expression after 6 h (50% ± 3%, *p* < 0.03, and 29% ± 4%, *p* < 0.0001, respectively, of the control). An immunohistochemical analysis of LRP1 and LRP2 protein expression in growth cones suggested that protein expression was also decreased by 50–60% after 6 h of culture in the presence of LRP siRNA (data not shown). Cultures treated with siRNA were then subjected to the growth cone turning assay.

To ensure that chemoattraction elicited by MTII was due to an MTII-LRP interaction, we first measured growth cone turning in response to MTII in the presence of the pan-LRP receptor inhibitor RAP. RAP was added to the culture medium 30 min prior to imaging, and growth cones were subsequently exposed to a microgradient of MTII. The addition of RAP abolished the turning effect elicited by MTII (−0.6°± 1.2°, *p* = 0.0015 *cf*. control, −1.8°± 1.1°; Fig. 2*C*). Because RAP was suspended in a 1:1 TBS solution, TBS was added to the medium alone as a control but was found to have no effect on the turning response elicited by MTII (data not shown). These findings suggest that LRP receptors are capable of mediating chemoattraction, but it does not indicate which MTII receptor, LRP1 and/or LRP2, is required.

Knockdown of LRP1 and LRP2 demonstrated that both LRP receptors are capable of mediating growth cone turning toward MTII (Fig. 2*C*). LRP1 knockdown abolished turning toward the MTII microgradient (3.5° 1.9°, *p* = 0.003, Fig. 2*C*). Similarly, turning in response to MTII after LRP2 knockdown was also abolished (3.6° ± 2.6°, *p* = 0.03, Fig. 2*C*). In comparison, turning toward MTII remained robust in the presence of the control siRNA (12.9° ± 2.7°, Fig. 2*C*). Simultaneous knockdown of both LRP1 and LRP2 also abolished the growth cone turning response to MTII (0.6° ± 1.5°, *p* = 0.002, Fig. 2*C*). To ensure that the reduced expression of LRP1 and LRP2 did not have any nonspecific effect on growth cone navigation, we examined growth cone turning in response to netrin-1 after dual knockdown of LRP1 and LRP2. There was no difference in growth cone turning in response to netrin-1 after knockdown of LRP1 and LRP2 compared with cultures transfected with control siRNA (Fig. 2*C*). These data clearly demonstrate that LRP1 and LRP2 are a novel class of axon guidance receptors capable of mediating growth cone attraction toward a source of MTII.

As axon guidance receptors, we would predict LRP1 and LRP2 expression to be biased to the turning side of the growth cone. We used rapid fixation during growth cone turning to demonstrate the dynamic response of LRP1 and LRP2 to a microgradient of MTII. We found that, as growth cones turn toward a source of MTII, both LRP1 and LRP2 expression were distributed asymmetrically, localized preferentially to the turning side of the growth cone (Fig. 2, *D* and *E*). Taken together, the data suggest that LRP1 and LRP2 do mediate growth cone chemotaxis and that the receptors are actively recruited or up-regulated at the membrane closest to the MTII gradient.

##### MT-LRP-mediated Chemoattraction Is a Calcium-dependent Process

**FIGURE 3.**
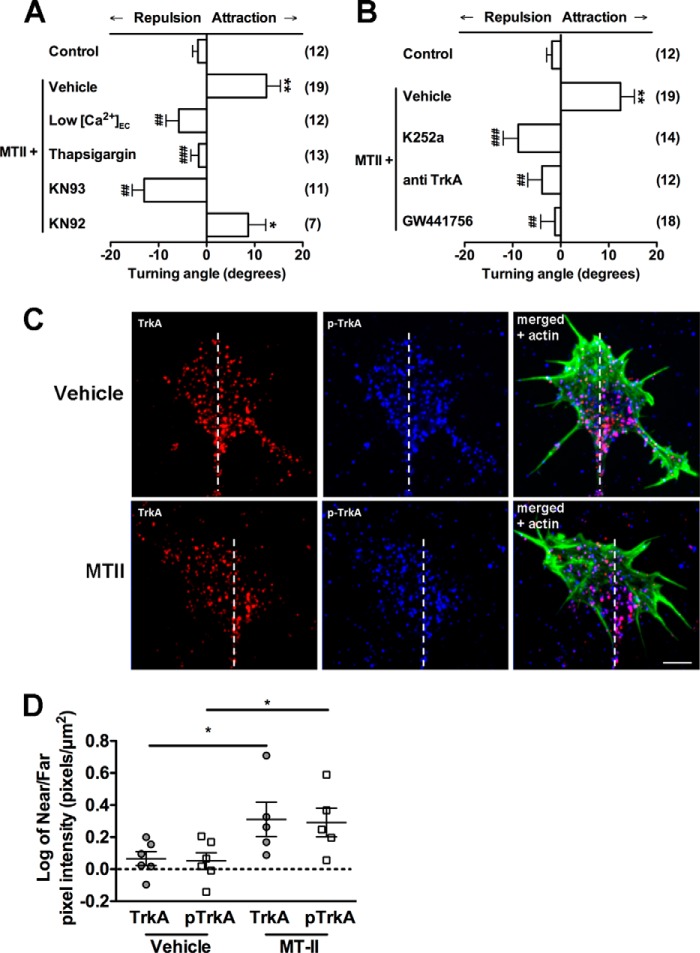
**MTII-LRP-mediated chemoattraction requires the activation of calcium signaling and co-receptors within the growth cone.** *A*, reducing the concentration of extracellular calcium (low [*Ca*^*2*+^]*_EC_*) reversed growth cone turning in response to MTII so that growth cones were repulsed by a microgradient of MTII. Depletion of intracellular calcium stores with thapsigargin abolished turning in response to MTII. The inhibitor of CaMKII, KN93, reversed turning in response to MTII so that growth cones were repulsed by a microgradient of MTII, whereas the inactive analogue KN92 had no effect on turning. *B*, inhibition of TrkA was shown to abolish growth cone turning in response to MTII. Inhibition of TrkA and other kinases by K252a reversed turning from attraction to repulsion. Specific inhibition of TrkA with GW441756 or a TrkA antibody abolished turning in response to MTII so that the turning angle did not differ from random control growth. *C*, representative immunocytochemistry images of individual growth cones turning in response to microgradients of vehicle (PBS) or MTII. Growth cones were rapidly fixed during turning and stained for TrKA (*red*) or phosphorylated TrKA (*pTrKA*, *blue*) and actin (*green*). The actin labeling was used to depict the growth cone area, and the growth cones were divided into near and far regions with respect to the micropipette for pixel intensity analysis. The *dotted line* drawn from the axon through the growth cone separates the near and far regions of the growth cone. *D*, quantification of total TrkA and phosphorylated TrkA expression localized to the near *versus* far side of the growth cone while turning toward a gradient of MTII. *** and ###, *p* < 0.0001; ** and ##, *p* < 0.001; * and #, *p* < 0.01. * represent data compared with the control; # represent data compared with MTII + vehicle.

Many axon guidance cues elicit a growth cone turning response by activating calcium signaling pathways (27). In the presence of low extracellular calcium, LRP-MTII-mediated turning was abolished to random control levels, with a trend toward chemorepulsion (−8.1° ± 3.8°, Fig. 3*A*). These data suggest that extracellular calcium is required for MTII-LRP-mediated growth cone turning. To determine whether intracellularly stored calcium was required, thapsigargin was applied to the bath 30 min prior to the start of the growth cone turning assays. Thapsigargin used in this manner depletes intracellular stores of calcium (28). The addition of thapsigargin abolished growth cone turning toward MTII, confirming that intracellularly stored calcium is required (−1.652° ± 1.620°, *p* < 0.0002, Fig. 3*A*). Taken together, these data suggest that LRP1- and LRP2-mediated chemoattraction requires both intracellular and extracellular calcium.

Axon guidance cues that are calcium-dependent signal through the calcium binding protein calcium/calmodulin-dependent kinase II (CaMKII) (29). To confirm that CaMKII activation is required for MTII-LRP-mediated chemoattraction, the CaMKII inhibitor KN93 was added to cultures 30 min prior to imaging. Inhibition of CaMKII reversed the chemotactic response to MTII and vehicle (from 12.44° ± 2.9° to −10.4°± 2.0°, *p* < 0.0001, Fig. 3*A*). KN92, the inactive analogue of KN93, was used as a control and had no discernible effect on the turning response (Fig. 3*A*).

##### MT-LRP-mediated Chemoattraction Activates Co-receptors Such as TrkA

LRP1-mediated neurite outgrowth has been shown previously to require the activation of TrkA (30). We initially used the general inhibitor K252a, which is known to inhibit TrkA, as well as other serine/threonine kinases, including PKA (31). Turning in response to MTII-LRP activation was reversed by the addition of K252a to the culture medium 30 min prior to imaging (Fig. 3*B*). To find out whether K252a action on TrkA and/or PKA was necessary for LRP-mediated chemoattraction, we used two approaches to directly target TrkA: We bath-applied an antibody directed at the extracellular epitope of TrkA as cells turned toward a gradient of MTII. The bath application of the antibody abolished turning so that turning was no longer different from a random control (Fig. 3*B*). We also used the specific TrkA pharmacological inhibitor GW441576 (31). This similarly abolished growth cone turning in response to MTII to control levels (Fig. 3*B*). If TrkA was being activated by MTII-LRP signaling, then we would expect an increase in phosphorylated TrkA in the growth cone during turning. To assess this, growth cones were exposed to the MTII gradient, and, after 15 min of turning, the growth cones were rapidly fixed and stained for total TrkA and phosphorylated TrkA (pTrkA) immunocytochemistry. The amount of TrkA and pTrkA on the side of the growth cone closest to the gradient, that is, the near, motile side of the growth cone, was assessed and compared with the far side of the growth cone (Fig. 3*C*). There was a significant increase in total and phosphorylated TrkA (Fig. 3*D*) on the near side of the growth cone. Taken together, our data support the hypothesis that signaling via LRP1 and LRP2 is calcium-dependent and that it is likely that the LRP proteins interact with multiple receptors, including TrkA, to effect growth cone guidance.

##### MTII Can Enhance Nerve Regeneration in Vivo

**FIGURE 4.**
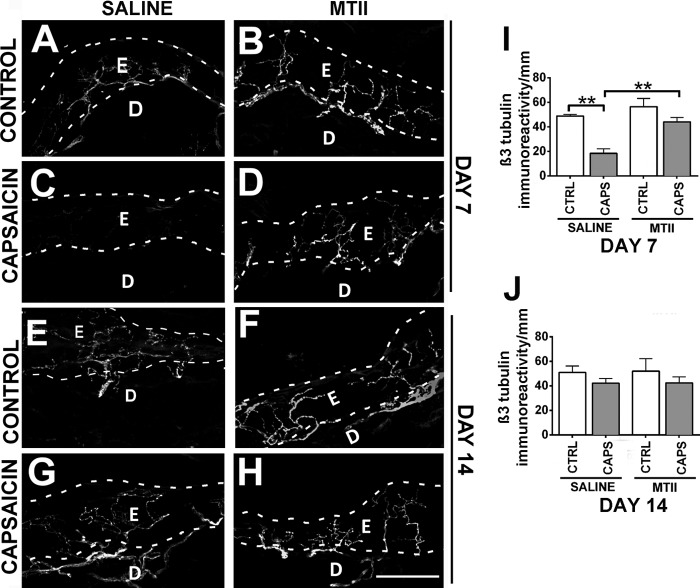
**MTII enhances regeneration in an *in vivo* chemical model of peripheral neuropathy.** *A* and *B*, rat skin treated with vehicle cream (control) showed no difference in the density of epidermal nerve fibers treated with saline (*A*) or MTII (*B*) at 7 days. *E*, epidermis; *D*, dermis. *C*, the density of epidermal nerve fibers was greatly reduced in rat skin treated with capsaicin cream and saline at 7 days. *D*, treatment with MTII restored epidermal nerve fiber density at 7 days. *E* and *F*, rat skin treated with vehicle cream (control) showed no difference in the density of epidermal nerve fibers treated with saline (*E*) or MTII (*F*) at 14 days. *G* and *H*, rat skin treated with capsaicin cream showed no difference in the density of epidermal nerve fibers treated with saline (*G*) or MTII (*H*) at 14 days. *Scale bar* = 50 μm (applies to *A–H*). *I*, quantification of epidermal nerve fiber density at 7 days. *CTRL*, control; *CAPS*, capsaicin. *J*, quantification of epidermal nerve fiber density at 14 days. **, *p* < 0.01; *, *p* < *0.05*.

To determine whether MTII-LRP signaling could serve as a therapeutic target in neuropathy and denervation, we used an *in vivo* model of nerve damage with chemically induced denervation of the skin. Capsaicin is an agonist of the vanilloid type 1 transient receptor potential cation (TRPV1) channels, key mediators of pain in C-fiber neurons, that predominate within the epidermis (32). Topical capsaicin treatment has been shown to cause a reversible retraction of TRPV1+ epidermal nerve fibers (ENFs), which, in humans, regenerate after 50–100 days (32), and is a validated experimental model of denervation (32). Treatment of rat dorsum skin with 8% capsaicin cream caused robust degeneration of the ENFs from the epidermis within 1 h of treatment (data not shown), and this degeneration persisted for a week (48.4 ± 1.5 ENF immunoreactivity/mm in control tissue compared with 18.4 ± 3.8 ENF immunoreactivity/mm in capsaicin-treated tissue, Fig. 4, *C* and *I*). Weekly biopsies were harvested to assess the effect of MTII on ENF regeneration after capsaicin-induced denervation (Fig. 4, *A–H*). By 14 days, regeneration was complete in saline-treated capsaicin tissue. Saline-treated, capsaicin-treated skin had an ENF density of 42.1 ± 4.0 ENF immunoreactivity/mm compared with saline-treated control skin (49.6 ± 1.5 ENF immunoreactivity/mm) (Fig. 4, *G* and *J*).

In contrast, intradermal injections of MTII enhanced regeneration so that ENF innervation of the epidermis was indistinguishable from the control at 7 days (Fig. 4, *D* and *I*). At this time point, capsaicin-denervated skin treated with MTII had an average ENF density of 45.0 ± 4.9 ENF immunoreactivity/mm compared with control skin treated with saline (48.4 ± 1.5 ENF immunoreactivity/mm). These results demonstrate the potent effect of MTII in chemically induced denervation. Importantly, there did not appear to be any excessive branching of ENFs in response to MTII in control tissue (Fig. 4, *B* and *I*). At 14 days, there were no differences between any of the treatments, confirming that MTII treatment had restored ENF innervation and had not caused excessive sprouting (Fig. 4, *E—H* and *J*). These data suggest that LRP-MTII-mediated chemoattraction could be an effective therapeutic in the restoration of innervation after injury or disease.

## Discussion

The failure of the peripheral nervous system to accurately and completely regenerate after injury results in significant morbidity. Injury or disease of peripheral nerves (neuropathy) may result in the loss of motor, sensory, and autonomic functions or development of debilitating neuropathic pain distal to the injury site (33). These symptoms are common because nerve regeneration after injury is often poor, absent, or aberrant (34). Understanding the mechanisms by which axons can regenerate and are guided to appropriate targets may improve our ability to enhance peripheral nerve regeneration and functional outcomes after nerve injury arising from either mechanical or metabolic/toxic insult. Chemotactic axon guidance has an essential role in normal development and is pivotal for accurate reinnervation of target tissues (35). Here we report a novel role for the lipoprotein receptors LRP1 and LRP2 in axon pathfinding and establish that this is a calcium-dependent process. The results indicate that LRP1 and LRP2 form a signaling complex that can regulate axon guidance *in vitro*, and, importantly, the only chemoattractant LRP ligand identified, MTII, is able to promote the regeneration of nerve fibers following capsaicin-induced denervation in rat skin *in vivo*.

The low-density lipoprotein receptor family has been implicated in neuronal regeneration and correct wiring of the nervous system (3, 13). Therefore, we tested numerous LRP ligands for their chemotactic ability. The ligands chosen had all been previously linked to neuronal growth and/or survival: α2m, a well characterized ligand of LRP1, has been implicated in neuronal signal transduction and shown to enhance neuronal growth *in vitro* (36, 37). ApoE3, a ligand of both LRP1 and LRP2, has been implicated in development, cognition, learning, and memory and is known to promote neurite extension in DRG neurons *in vitro* (9, 38, 39). Tissue-type plasminogen activator is a known ligand of LRP1 with a putative role in plasticity and neuromodulation of long-term potentiation (8). MTII, a ligand of both LRP1 and LRP2, has a role in neuroprotection, neurite outgrowth, and nerve regeneration *in vivo* (16, 18, 22, 40). MTII has strong growth-promoting properties (7), whereas MTIII displays growth inhibitor properties (23). MTIV is found predominately in the skin and is not known to have a neuronal role (41). Vitamin D/vitamin D binding protein complexes interact with LRP2 and have been postulated to be important for cognition (42). Transthyretin has been shown to mediate neuroprotection and neurite extension via LRP2 (43). RAP is a chaperone ligand of the LRP-receptor family and is an established pan-LRP-competitive inhibitor (44). Of these LRP ligands, three were chemorepulsive: α2m, tissue plasminogen activator, and MTIII. Conversely, MTII was the only LRP ligand found to be chemoattractive.

We demonstrated that MTII-induced chemoattraction required both LRP1 and LRP2 signaling. Growth cones with reduced LRP1 or LRP2 expression no longer turned toward the microgradient of MTII. The expression of LRP1 and LRP2 on growth cone membranes preferentially localized to the turning, or navigating, side of the growth cone, suggesting an activation or recruitment of the receptors by MTII. Furthermore, the close physical association suggests that LRP1 and LRP2 may form complex signaling domains, interacting with each other and other co-receptors. Our data as well as those of others (30) would suggest that such signaling complexes include LRP1, LRP2, and other receptors, such as the nerve growth factor receptor TrkA, and that these receptor complexes are actively recruited to the motile, navigating side of the growth cone. The potential for LRP1-LRP2 interaction has been alluded to previously. For example, it has been shown that MTII could bind LRP1 as well as LRP2, but the biological role of the MTII-LRP1 interaction was unclear (18). LRP1 and LRP2 potentially interact in the sonic hedgehog signaling pathway. The protease nexin 1 interacts with LRP1 to antagonize sonic hedgehog signaling (10), whereas, conversely, sonic hedgehog can be activated by LRP2 signaling (45). These previous findings support the notion of LRP1/LRP2 receptor cross-talk. Our data extend these earlier studies and demonstrate that both LRP1 and LRP2 receptors are necessary to direct axon guidance in response to MTII *in vitro*.

**FIGURE 5.**
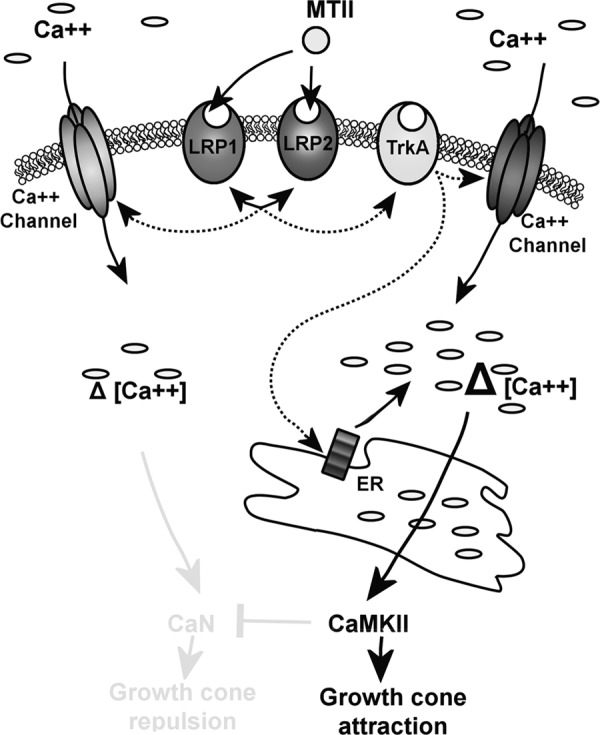
**Proposed signaling pathway for MTII-LRP chemotaxis.** MTII binds LRP1 and LRP2, which engage in receptor cross-talk, recruiting TrkA. This pathway likely activates Ca^2+^ channels and release of Ca^2+^ from internal stores such as the endoplasmic reticulum, resulting in a large change in intracellular Ca^2+^ levels. This would activate CaMKII and PKA and culminate in growth cone attraction. In the event of a loss of LRP1 or LRP2, the receptor cross-talk cannot occur, and, therefore, there is no growth cone turning. If TrkA is inhibited, then it is likely that a small influx of Ca^2+^ occurs through a TrkA-independent LRP1/2-dependent process, activating calcineurin and the repulsive pathway.

Calcium signaling was required for LRP transduction of chemotactic cues to effect growth cone navigation. Calcium signaling is downstream of most guidance cues (27). Spatial and temporal fluxes of intracellular calcium in the growth cone are able to trigger both attraction and repulsion (46). This is in part due to the activation of differentially sensitive calcium-dependent binding proteins such as calcineurin and CaMKII (29). Calcineurin mediates growth cone repulsion. Conversely, CaMKII activation results in attraction (29). We found that both intracellular and extracellular calcium sources were required for LRP-mediated chemotaxis. The reduction of extracellular calcium or inhibition of downstream calcium effectors such as CaMKII resulted in the reversal of the growth cone response to MTII from attraction to repulsion. This reversal is consistent with the MTII activating the calcium-dependent calcineurin/CaMKII switch reported previously (29). We propose a model by which signaling hubs between LRP1, LRP2, and other receptors, such as TrkA, activate calcium signaling pathways that regulate growth cone motility and, therefore, chemotaxis (Fig. 5).

The finding that MTII-mediated LRP1/2 signaling activates growth cone chemoattraction *in vitro* suggests that MTII may be useful in a therapeutic context *in vivo*. Axonal chemotaxis is a functionally important component of neuronal regeneration because regrowth must be directed to the lesion site so that appropriate connections are reformed (35). Although the environment of the regenerating neuron is inherently less permissive than that of a developing neuron, the cellular processes that encompass neurite regeneration are thought to recapitulate many aspects of neurite outgrowth in development (reviewed in Ref. 47). This hypothesis lends itself to the notion that the chemoattractive property of MTII observed in developing sensory neurons *in vitro* may also be applicable to axons that resprout during post-injury regeneration *in vivo*.

We used the capsaicin model of peripheral denervation to assess the ability of MTII to effect axonal regeneration. Quantifying the ENF density after capsaicin treatment using a method described previously (48), we demonstrated that capsaicin application resulted in a robust and predictable denervation, with regeneration in control or saline-treated capsaicin skin complete within 14 days. Treatment with MTII enhanced this regeneration so that ENF regrowth into the epidermis was complete in only 7 days. Although quantification in this study was limited to skin sections that did not contain hair follicles, we found that MTII not only enhanced regeneration but, importantly, did not result in excessive sprouting above control levels. This finding was important, given that excessive sprouting is known to be detrimental, and thought to cause aberrant sensory perceptions, such as allodynia (49). Indeed, past clinical trials for neuropathy treatments have failed because of excessive regrowth of nerves and subsequent development of neuropathic pain (50). In this study, MTII was delivered *in vivo* by intradermal injection. To enhance the therapeutic potential of MTII in peripheral neuropathy, the delivery method needs to be refined to deliver a topical microgradient of MTII that can accurately redirect regenerating nerves back into the epidermis. Given that it is likely that both traditional and novel receptor-ligand systems are necessary to guide axon regeneration after nerve injury, the MTII-LRP axon guidance system offers a novel, context-dependent signaling mechanism with great promise as a therapeutic candidate in peripheral nerve damage.

The data described here shed new light on the function of LRP1 and LRP2 receptors as chemotactic receptors that have the therapeutic potential to guide regeneration *in vivo*. Taken together with existing data, our data suggest a putative role for LRPs as neurotrophic-like receptors; that is, to promote the survival, neurite outgrowth, and chemotactic guidance of axons (7, 17, 18, 22, 35).

## Author Contributions

L. M. L. performed experimental work and data analysis and contributed to the conception of experiments and writing of the manuscript. M. P. and L. S. B. contributed to experimental work. R. G. contributed to the conception of experiments. B. V. T., A. K. W., and L. F. conceived experiments. All authors contributed to the writing of the manuscript.

## Footnotes

3 The abbreviations used are:

LRP: low-density lipoprotein receptor-related protein receptor
tPA: tissue plasminogen activator
MT: metallothionein
DRG: dorsal root ganglion/ganglia
RAP: receptor-associated protein
VD/VDBP: vitamin D/vitamin D binding protein complex
CaMKII: calcium/calmodulin-dependent kinase II
ENF: epidermal nerve fiber
α2m: α-2-macroglobulin.
